# SLDR: a computational technique to identify novel genetic regulatory relationships

**DOI:** 10.1186/1471-2105-15-S11-S1

**Published:** 2014-10-21

**Authors:** Zongliang Yue, Ping Wan, Hui Huang, Zhan Xie, Jake Y Chen

**Affiliations:** 1 Institute of Biopharmaceutical Informatics and Technology, Wenzhou Medical University, Wenzhou, Zhejiang Province, China; 2 School of Informatics, Indiana University, Indianapolis, IN 46202, USA; 3 Indiana Center for Systems Biology and Personalized Medicine, Indiana University, Indianapolis, IN 46202, USA; 4 Bioinformatics lab, Capital Normal University, Beijing, China

## Abstract

We developed a new computational technique called **S**tep-**L**evel **D**ifferential **R**esponse (SLDR) to identify genetic regulatory relationships. Our technique takes advantages of functional genomics data for the same species under different perturbation conditions, therefore complementary to current popular computational techniques. It can particularly identify "rare" activation/inhibition relationship events that can be difficult to find in experimental results. In SLDR, we model each candidate target gene as being controlled by *N *binary-state regulators that lead to ≤2^N ^observable states ("step-levels") for the target. We applied SLDR to the study of the GEO microarray data set GSE25644, which consists of 158 different mutant *S. cerevisiae *gene expressional profiles. For each target gene *t*, we first clustered ordered samples into various clusters, each approximating an observable step-level of *t *to screen out the "de-centric" target. Then, we ordered each gene *x *as a candidate regulator and aligned *t *to *x *for the purpose of examining the step-level correlations between low expression set of *x *(R_o_) and high expression set of *x *(R_h_) from the regulator *x *to *t*, by finding max *f*(*t, x*): |R_o_-R_h_| over all candidate × in the genome for each *t*. We therefore obtained activation and inhibitions events from different combinations of R_o _and R_h_. Furthermore, we developed criteria for filtering out less-confident regulators, estimated the number of regulators for each target *t*, and evaluated identified top-ranking regulator-target relationship. Our results can be cross-validated with the Yeast Fitness database. SLDR is also computationally efficient with o(N^2^) complexity. In summary, we believe SLDR can be applied to the mining of functional genomics big data for future network biology and network medicine applications.

## Introduction

Identifying novel gene regulatory relationship from large-scale functional genomics data has been a major theme for the characterization of complex biomolecular systems. Gene regulator identification can be identified from gene expression data using DNA microarrays. With tens of thousands of microarray experiments deposited into public databases for yeast, Drosophila, Arabidopsis, mice, and humans, one may reconstruct molecular interaction or regulation relationships from mining the data without conducting specific experiments to test whether a candidate regulator-target relationship exists. For example, James *et al *[1] examined temporal gene expression patterns during chondrogenic differentiation in a mouse micromass culture system. Then, they determined transcriptional regulation by observing the impact of changed expression of molecules onto changed gene functional categories. Lorenz *et al *[2] used microarray analysis and scale-free gene networks analysis to identify candidate regulators in drought-stressed roots of loblolly pine. Systematic approaches to reconstruct transcriptional modules and identify their perturbation conditions are under way [3]. Albert *et al *[4] summarized recent findings that the disruption of regulatory relationships may lead to human diseases, therefore shedding new light on disease intervention on gene regulatory relationships instead of genes as possible drug targets--hence the new field of "network medicine". These examples show a surging interest among genome biologists to study gene regulatory relationships.

Traditional experimental gene regulator finding methods, e.g., those using gene knockouts, synthetic lethality, or chip-seq in eukaryotes, are too costly to serve as the primary platform with which scientists explore the large combinatorial space between all candidate pairs of genes [5-7]. To overcome the data coverage gap, many computational methods have been proposed, e.g., homologous gene regulator database search, clustering of gene expression profiles and transcription factor binding site pattern matching, physics-based modeling of candidate transcription factors and target binding relationships, and network based methods [4,8]. For example, Ru-Fang Yeh *et al *[9] introduced an accurate and efficient technique that performs homologous gene regulator database search in higher eukaryotes to annotate gene regulators for the human genome. Stephane *et al *[10] developed a rigorous statistical test to establish a link between selection threshold of putatively regulators and the identified false positive genes in clusters of candidate gene targets derived from gene expression profiles. Gerhard *et al *[11] developed ANREP, a system that can identify exact pattern matches to motifs with spacing constraints and approximate matches recursively. Physics-based methods that characterize protein-ligand relationships, e.g., the MM-based methods, have also been proposed [12].

In this study, our aim is to develop a new computational method to identify **genetic regulatory relationships **that are difficult to uncover using previously reported techniques. This type of relationships differ from gene regulatory relationships in that genes in the former type may affect each other indirectly through other genes or molecular regulation mechanisms while genes in the latter type affect each other as direct, observable regulator-target relationships. Current databases often cover reasonably well highly-connected gene regulators, or "hubs" of gene regulations in the gene regulatory network [13]; however, for low-connectivity regulators, or "de-centric nodes" in the gene regulatory network, the coverage is often poor because the chance for randomly observing their activities is low. **S**tep-**L**evel **D**ifferential **R**esponse (SLDR) is a new computational method developed to identify de-centric genetic regulatory relationship candidates. The input of SLDR is the functional genomics data under permutated perturbation conditions. In SLDR, we specifically search for all qualifying target genes, each which is controlled by *N *binary-state regulators that lead to ≤2^N ^observable expression levels--which we call "step-levels"--of the target gene. The output of SLDR is statistically significant findings candidate genetic regulatory relationships. We describe our study in detail next.

## Method

### An overview of the framework

**Figure 1:**
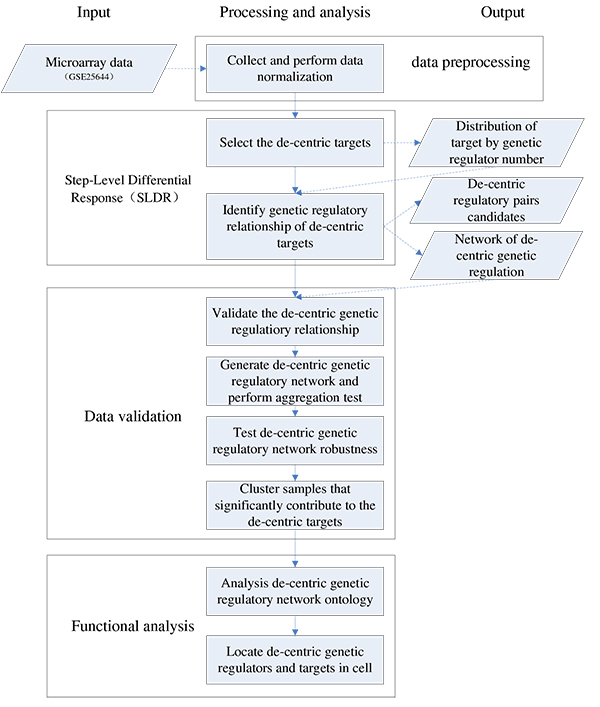
**An overview of the SLDR framework for identifying novel genetic regulatory relationships**. The input data is a microarray data set. The intermediate process were involved data normalization, Pearson Correlation Model, cross-validatation in Yeast Fit Database, network generation, aggregation test, hypergeometric test, robustness detection and Unweighted Pair Group Method with Arithmetic Mean (UPGMA) clustering. The output data are: a distribution curve of targets, two network of de-centric genetic regulatory relationship, and .txt or .csv files containing the activation/inhibition pairs of de-centric targets and the corresponding genetic regulatory networks.

We show an overview of workflow of the SLDR data analysis framework (Figure 1**)**. First, the expression values in perturbed gene expression data sets will be averaged across biological independent replicates and groups without mutation will be filtered off. Second, de-centric genetic regulatory relationship targets will be selected after clustering of gene expression profiles. Third, a statistical correlation-based model will be applied to the extraction of significant de-centric activation/inhibition relationship pairs and a threshold will be applied to the rejection of low-confidence de-centric genetic regulatory relationship pairs. Fourth, the de-centric genetic regulatory network thus generated will be assessed with network "index of aggregation" test. Fifth, novel candidate genetic regulatory pairs will be evaluated with several public gene regulation databases and a hyper-geometric test will be used to rank the top 10 suspected de-centric targets predicted by SLDR. Sixth, the robust of the de-centric networks will be tested by performing the shuffle method to introduce noise. Seventh, samples will be clustered, which is significantly contribute to the de-centric targets. Additionally, the de-centric networks' function will be analyzed by Gene Ontology and sub-cellular localization. The comprehensive output of SLDR analysis can consist of: a distribution curve of target by genetic regulator number, two networks of activation/inhibition de-centric genetic regulation, and a list of the top 10 suspected de-centric genetic regulatory pair candidates.

### 1. Preparation of functional genomics data

We used raw data GSE25644 from the Gene Expression Omnibus (GEO) database (http://www.ncbi.nlm.nih.gov/geo/) as the input. GSE25644 is a DNA microarray gene expression profile with all 158 viable protein kinase/phosphatase deletions in S. cerevisiae under a single growth condition [14]. Each mutant was profiled four times, from two independent cultures on dual-channel microarrays using a batch of wild-type (WT) RNA as a common reference. The GSE25644 was normalized by averaging each of the two independent cultures' results on microarrays, and before our algorithm was applied, the wild-type groups were filtered off.

### 2. Selection of the de-centric targets

**Figure 2:**
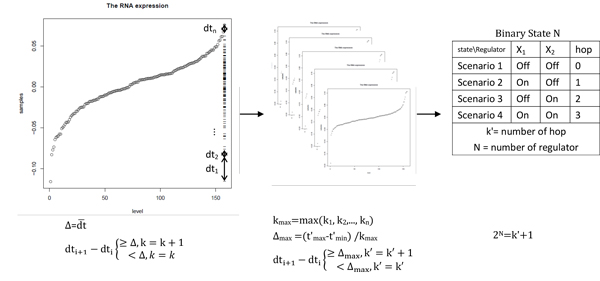
**The workflow of de-centric targets generation**. First, we used average changing value as standard to screen out the number of steps. Second, to normalize the steps, we use the largest steps gene as new standard to screen out the number of steps. Third, we apply binary states method to find out theoretical regulators' number according to the number of steps.

The selection of de-centric targets is based on clustering of gene expression profiles. To find the potential de-centric targets which be regulated with N regulators, we modeled k'+1≤2^N ^for each candidate target gene as being controlled by N binary-state regulators that lead to k' observable states ("step-levels"). Here, we introduced k' which is the number of steps generated from each gene RNA expression. If there are sufficiently large collections of functional genomics experiments, each being performed under a heterogeneous perturbation condition, the method will search each target's genetic regulator candidates to test if a significant switch-responder pattern exists before ranking candidate genetic regulators (Figure 2).

1) In order to find out the huge steps, we use average steps as a standard to filter. The average increasing value Δ of each gene was calculated and averaged values were sorted. The average increasing value Δ was calculated by the maximum value of the gene expression (we assume the target gene t) minus the minimum value and then divided by the number of samples n. Δ_t_=(t_max_-t_min_)/n.

2) The average increasing value Δ_t _of a target was regarded as a standard to seek steps which means the number of dj larger than Δ_t_, which we defined as k value. For each target, gene expression was sorted from low to high and then we calculated the difference between adjacent samples, dj. If the difference dj is smaller than corresponding Δt, k will be retained. The formula of step k is shown below:

$$\text{d}{\text{t}}_{\text{i}+1}-\text{d}{\text{t}}_{\text{i}}\left\{\begin{array}{c} \geq \text{Δ},k=k+1\\ <\text{Δ},k=k \end{array}\right)$$

3) Iteration was performed for every target to find out each k of targets. k values were sorted from low to high and the largest k_max _corresponds to target t'.

4) To avoid the situation that fake steps with small change causes high k' in individual, every target was normalized by the new average increasing value Δ_max _as a standard to seek for new step levels (k') of each target. The formulas of new average increasing value Δ_max _and step k' are shown below:

$${\text{Δ}}_{\text{max}}=\left({\text{t}}_{\text{max}}^{\prime }-{\text{t}}_{\text{min}}^{\prime }\right)/{\text{k}}_{\text{max}}\;{\text{t}}_{\text{i}+1}-{\text{t}}_{\text{i}}\left\{\begin{array}{c} \geq {\text{Δ}}_{\text{max}},k^{\prime }=k^{\prime }+1\\ <{\text{Δ}}_{\text{max}},k^{\prime }=k^{\prime } \end{array}\right)$$

5) The binary state N was calculated, and N means the number of genetic regulators of each target. For instance, assuming that a target's binary state N is 2, this target would have less than 3 steps within 4 step-levels theoretically. The formula of N binary-state is shown below:

$${\text{2}}^{\text{N}}={\text{k}}^{\prime }+\text{1}$$

We can use the cluster k' to calculate the theoretical number of genetic regulators of the de-centric targets.

### 3. Identification of genetic regulatory relationship among genes

**Figure 3:**
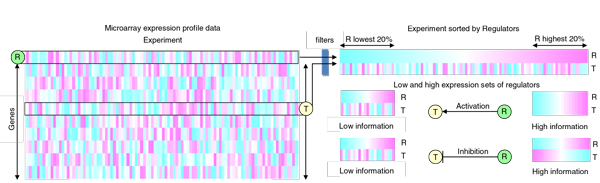
**The model of activation/inhibition predicted based on the expression pattern associated with Pearson Correlation**. A gene expression profile dataset was represented as a matrix, where columns indicated different samples and rows indicated different genes. The expression profiles of regulators were sorted, and the lowest 20% and highest 20% were regarded as low expression sets and high expression sets. The activation and inhibition of potential regulators had low information in low expression set and high information in high expression set.

The genetic regulatory relationships of regulators to targets were predicted based on the expression pattern associated with Pearson Correlation. First, the model of activation/inhibition is determined by the low gene expression pattern and the high gene expression pattern in (Figure 3). Here we sorted the expression values of every gene regarded as regulator, then align the other potential targets to it. The regulator's lowest 20% expression was regarded as low expression set, and highest 20% expression was regarded as high expression sets since all of the de-centric steps located in these expression sets. When the low expression set has low information and high expression set has high information, we supposed that a activation or inhibition existed. For instance, if there exists a positive genetic regulator activating its target, the correlation in low gene expression's step-level would be low to 0 and the correlation in high gene expression's step-level would be high to 1.

To explore the potential activation/inhibition genetic regulatory relationship between regulator × and target t, each gene t was regarded as a genetic target candidate was aligned to each step level of the regulator x. The low step levels of t_0 _and the high step levels of t_h _were used to compute Pearson correlation R_0 _and R_h _respectively_. _The Pearson correlation formula is shown below:

$$\rho \left(\text{x},\text{t}\right)=\frac{\text{cov}\left(\text{x},\text{t}\right)}{\rho \text{x}\rho \text{t}}=\frac{\text{E}\left[\left(\text{x}-\mu_{\text{x}}\right)\left(\text{t}-\mu_{\text{t}}\right)\right]}{\rho \text{x}\rho \text{t}}$$

Where, cov is the covariance between potential regulator × and target t, **ρ**x is the standard deviation of x, **µ**_x _is the mean of x, and E is the expectation.

In order to get the strict result of inhibition and activation relationship, the high information and low information were designed as 0.8 and 0.2. For instance, the R_o _located in (-0.2,0.2) and the R_h _located in the (0.8,1) indicated the × has positive genetic regulation on t. For illustrative purposes, we show a simple table (Table 1) to explicitly explain the activation/inhibition models of the genetic regulator × to target t.

**Table 1:** Representation of Activation/Inhibition genetic relationship pairs identified and the threshold used between R_0 _and R_h _cases.

	R_0_	R_h_
x→(+)t	→0(-0.2,0.2)	→1(0.8,1)
x→(-)t	→0(-0.2,0.2)	→-1(-0.8,-1)

To reject the incorrect genetic targets t, we required that the middle gene expression step-levels of the genetic targets t shouldn't have a significant larger change than the average change values of gene expression. Assuming that one genetic target t has one middle gene expression step-level from step i to step j and the lowest gene expression value among t_i _to t_j _is t_low_, the change of gene expression from t_n _(n in i to j) to t_low _shouldn't be more than the genetic regulator's average change level of the value Δ_t_. The formula of rejection criteria is shown below:

$${\text{t}}_{\text{n}}-{\text{t}}_{\text{low}}<{\text{Δ}}_{\text{t}}*\left(\text{j}-\text{i}\right)\text{n}\in \left(\text{i},\text{j}\right)\text{while}\;{\text{Δ}}_{\text{t}}=\left({\text{t}}_{\text{max}}-{\text{t}}_{\text{min}}\right)/\left({\text{t}}_{\text{size}}\right)$$

To examine the step-level correlation between low step levels of the *x *(R_0_) and high step levels of the *x *(R_h_) between the *x *and *t*, all genetic regulator candidates were ordered to each target using max *f*(*t, x*): |R_o_-R_h_|. Then we choose top 10 high |R_o_-R_h_| genetic regulatory pairs, since the number of nodes generating the genetic regulatory network should be balanced and moderate to present the de-centric targets. If the pairs are strict, they will not organize a network with enough pairs. If the pairs are relax, they will introduce noise of weak links in the networks.

### 4. Generation of the de-centric genetic regulatory network and testing of network significance

We generated the de-centric genetic regulatory network by the top 10 high |R_o_-R_h_| genetic regulator pairs and performed statistical data analysis tests to detect the significance of the connected network. Our hypothesis of this statistical evaluation is that if the prediction model indeed consists of de-centric targets involved in the same process even if complex and broad, then we should expect that the connectivity among the de-centric targets be lower than the connectivity among a set of randomly selected genes.

We defined the index of aggregation of a network [8] as the ratio of the size of the largest sub-network that exists in this network to the size of this network. Note that the size is calculated as the total number of genes within a given network/sub-network.

To test the hypothesis that the predicted targets are less connected than a randomly selected set of targets, we developed the null hypothesis test using the following re-sampling procedure :

1) Randomly select from the pool of genetic regulators to targets, the same number of predicted targets generated from our method.

2) Retrieve the top 10 genetic regulators of each random target using |R_o_-R_h_| criteria.

3) Compute the index of the aggregation of superset.

4) Repeat steps 1 through 3 for 500 times to generate a distribution of the index of aggregation under random selection.

5) Compare the index of aggregation from our method with the distribution obtained in step 4 and calculate the p-value.

### 5. Significance testing of the de-centric genetic regulatory relationships

Our result is validated in the Yeast Fitness Database (http://chemogenomics.med.utoronto.ca/fitdb/fitdb.cgi). FitDB is a searchable database of quantitative chemical-genetic interactions based on data in Hillenmeyer [15]. A gene search allows viewing of the compounds that are most sensitive to the gene specified in a heterozygous and homozygous yeast deletion strain, including a view of yeast deletion strains that behave similarly to the gene of interest. Compounds can also be searched to identify heterozygous or homozygous deletion strains exhibiting hypersensitivity to compound, including a view of compounds that behave similarly to the compound of interest.

Hypergeometric test was introduced to see the significant of the top 10 high |R_o_-R_h_| pairs in genetic regulator candidates. The validated number of the top 10 high |R_o_-R_h_| pairs in Yeast Fit database was k. The total number of the top 10 high |R_o_-R_h_| pairs is n. The validated number of randomly chosen pairs in Yeast Fit database is K. The total number of randomly chosen pairs is N. The use of hypergeometric test is illustrated in (Table 2).

**Table 2:** A contingency table showing how to perform the hypergeometric test

	top 10	random
validated	*k*	*K*
un-validated	*n *− *k*	*N-K*
total	*n*	*N*

The variable number of top 10 high |R_o_-R_h_| pairs × follows the hypergeometric distribution by its probability mass function (pmf) given by the formula below:

$$\text{P}\left(\text{X}=\text{k}\right)=\frac{\left(\begin{array}{c} \text{K}\\ \text{k} \end{array}\right)\left(\begin{array}{c} \text{N}-\text{K}\\ \text{n}-\text{k} \end{array}\right)}{\left(\begin{array}{c} \text{N}\\ \text{n} \end{array}\right)}$$

### 6. Test de-centric genetic regulatory network robustness

In order to detect the robustness, we introduced the noise on the gene expression profiles of the 158 viable protein kinase/phosphatase deletions strains. The noise was designed as increasing 5%, 10%, 15%, 20%, 30%, 50%, 70% of noise by randomly shuffling the expression values of each sample.

### 7. Cluster the samples that significantly contribute to the de-centric target

The 158 viable protein kinase/phosphatase deletions' profiles was clustered by UPGMA (Unweighted Pair Group Method with Arithmetic Mean). The agglomerative clustering method UPGMA is one of the most popular methods for the classification of sampling units on the basis of their pairwise similarities in relevant descriptor variables. We used UPGMA algorithm to construct a rooted tree that reflects the structure in a pairwise samples' similarity matrix.

At each step, the nearest two clusters are combined into a higher-level cluster. The distance between any two clusters A and B is taken to be the average of all distances between pairs of objects "x" in A and "y" in B, that is, the mean distance between elements of each cluster:

$$\frac{1}{\left|A\right|\times \left|B\right|}\underset{x\in A}{\sum }\underset{y\in B}{\sum }d\left(x,y\right)$$

Then we find a minimal threshold in the hierarchical tree and pick a representative cluster. Delete it to see the effect of the cluster on finding of de-centric targets.

### 8. Analysis de-centric genetic regulatory network ontology

In order to explore the function of the de-centric regulatory networks, ClueGo [16], a Cytoscape plug-in was used. ClueGO performs single cluster analysis and comparison of clusters. From the ontology sources used, the terms are selected by different filter criteria. The related terms which share similar associated genes can be fused to reduce redundancy. The ClueGO network is created with kappa statistics and reflects the relationships between the terms based on the similarity of their associated genes. ClueGO charts are underlying the specificity and the common aspects of the biological role. The significance of the terms and groups is automatically calculated.

### 9. Locate the de-centric regulators and targets in cell

The sub cellular location of de-centric regulators and targets were retrieved in the Comprehensive Yeast Genome Database [17], which derived from experiments, (CYGD: http://mips.gsf.de/genre/proj/yeast/) and display by the tool, Cerebral, a cytoscape plug-in. Cerebral uses sub cellular localization attribute to create a layered view of a cell, placing nodes in the region of the screen corresponding to the appropriate localization. [18]

## Results

### 1. Determination of de-centric genetic regulated targets from the processed data

**Figure 4:**
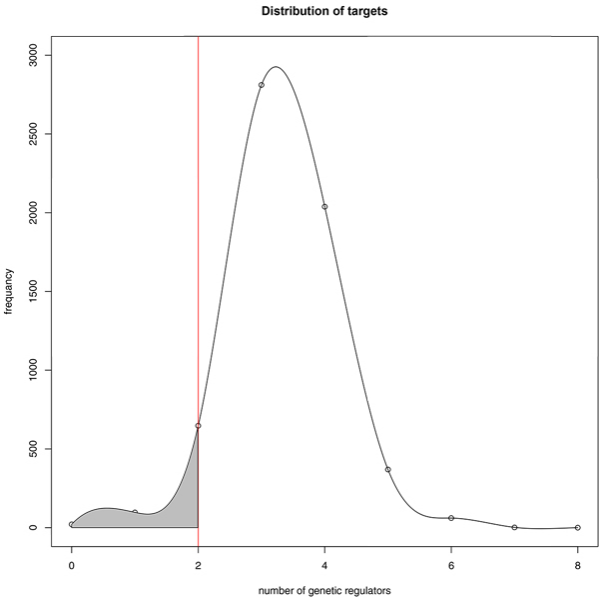
**A histogram showing the distribution of regulator counts for all data in the Yeast Fit Database**. The red line stands for the genetic regulators equal 2 within step levels k' from 2 to 3. The shade area presents the genetic regulators equal or below 2 step levels excluding 0 within k' from 1 to 3.

The distribution of the number of genetic regulators for each target follows Gaussian distribution which means the number of genetic regulators is a random variable independently drawn from the same distribution. 453 targets were selected, which has no more than two genetic regulators after the clustering of gene expression profiles. (Figure 4) According to the formula: 2^N ^= k'+1, the N was determined by the cluster k' and all targets were clustered into 8 groups with N varying from 0 to 7. If the N was chosen as 1, which means the theoretical target's genetic regulator equal to 1, the thresholds was so strict that include 59 out of 6047 targets with coverage 1%. If the N was chosen as 3, the thresholds was so relax that include 2579 out of 6047 with coverage 43% nearly to half. Meanwhile, the mis-clustering will be severe with k' varied from 1 to 7, which will be discussed afterwards. We chose the modest thresholds; N as 2 that include 453 out of 6047 with coverage 7.5% as shown in left shade region separated by red line which displayed the target's genetic regulator equal or below 2 excluding 0.

The 2^N ^= k'+1 is the key point to control the theoretical genetic regulator number of targets. Especially the selection of binary-state N determines the theoretical number of genetic regulators directly. The reason we call N theoretical genetic regulator number is that in the formula 2^N ^= k'+1, we cannot be certain about the number of N when several genetic regulator have same changing DNA expression level on their target giving merged step-levels. Assuming that a target t have n genetic regulators x_1_, x_2 _... x_n_. And f(x_1_, x_2 _... x_n_) stands for step levels due to the combined genetic regulatory effect on t. The states of × can be either 1 or 0 to indicate whether × is activated or not. The number of target's step-levels f_t_(x_1_, x_2 _... x_n_) is greater than n: f_t_(x_1_, x_2 _... x_n_) ≥ n + 1. For instance, if x_1_, x_2_, x_3 _have similar regulation ability on t, the f(1,0,0), f(0,1,0), f(0,0,1) will give a merged level. Similarly the f(0,1,1), f(1,0,1), f(1,1,0) will give another merged level. In this case, the step levels of t is 4 = n+1 that stands for four levels of f(0,0,0), f(1,0,0), f(1,1,0) and f(1,1,1). Hence, the N will be 2 meaning that our predicted targets of 2 genetic regulators will contain some targets of 3 genetic regulators in extreme case. And the mis-clustering will be more severe when applied for prediction of targets with more genetic regulators.

### 2. Identification of activation/inhibition genetic regulatory relationship details

According to the Pearson Correlation model of Activation/Inhibition, 89 targets were selected with 3473 activation pairs in initial 453 de-centric targets generated from SLDR and 94 targets were selected with 2271 inhibition pairs in initial 453 de-centric targets generated from SLDR. After applying the criteria of rejection, we found 83 targets with 3190 activation pairs and 93 targets with 2128 inhibition pairs. After ordering all 5318 regulation pairs in the genome for each target by finding max *f*(*t, x*): |R_o_-R_h_| and choosing top 10 high pairs, 610 activation pairs (Additional file 1) and 494 inhibition pairs (Additional file 2) were selected. 176 targets candidates were queried in Yeast Fitness Database, 64 targets were identified afterwards. Among them, 33 targets with 115 activation pairs and 31 targets with 97 inhibition pairs were validated. (Table 3)

**Table 3:** Summary Statistics for validated de-centric targets and genetic regulatory relationship identified

	Activation			inhibition		
	**Validated**	**total**	**rate**	**validated**	**total**	**rate**

pairs	115(33)	610(83)	19%(40%)	97(31)	494(93)	20%(33%)

**Figure 5:**
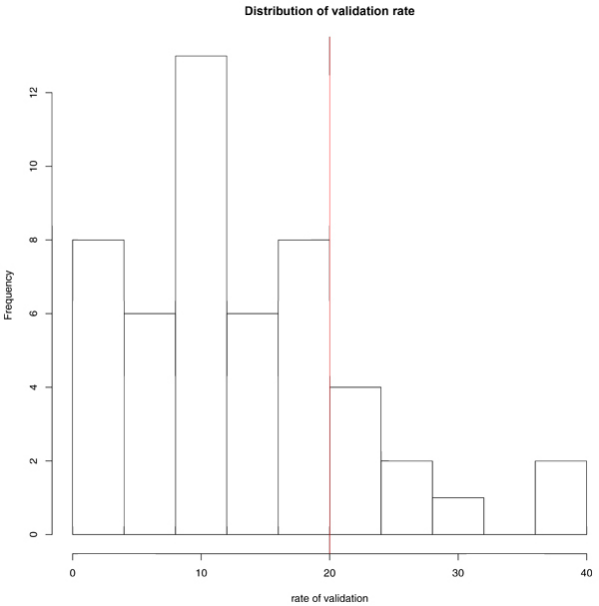
**The distribution of validation rate of randomly chosen pairs**. The red line drawn at rate of validation = 20 refers to a 20% validation ratio for top 10 |R_o_-R_h_| pairs. 9 out of 50 is above to 18%, 41 out of 50 is below 82%.

The contingency table of genetic regulatory relationship network is shown in (Table 4**)**. The top 10 high |R_o _-R_h_| genetic regulatory pairs is 1104 within 212 pairs been validated. The randomly chosen pairs is 7389 within 433 pairs been validated. The p-value of 7.44e^-43 ^showed the significant difference between top 10 |R_o_-R_h_| pairs and randomly chosen pairs. We also preformed resample of randomly chosen pairs (the same size to 10 |R_o_-R_h_| pairs: 1104) with Pearson Correlation cut off 0.2 and 0.8 for 50 times and generated the distribution of validation rate (Figure 5). 18% of pairs above 20% validation rate indicated that the top 10 |R_o_-R_h_| pairs is more significant than randomly one.

**Table 4:** A distribution of significant genetic regulatory relationship according to rank groups.

	top 10	Random
**validated**	212	433
**un-validated**	892	6956
**total**	1104	7389

**Figure 6:**
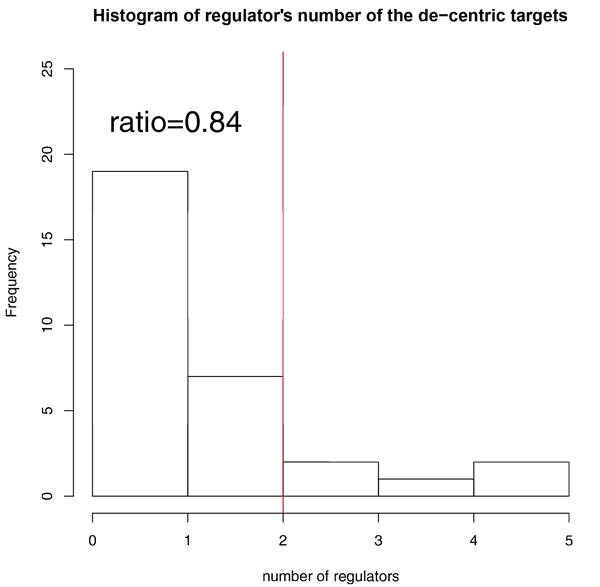
**A histogram showing the distribution of regulator counts among all the de-centric gene targets**. A red line shows the ratio of the target's number which regulators' number equal or below 2 to the total target's number is 0.84.

The 174 de-centric genetic regulatory targets (81 active targets, 91 inhibited targets, 2 in both active targets set and inhibited targets set) predicted by SLDR were queried in the Yeastract Database (http://www.yeastract.com/index.php). The evidence of DNA binding and expression in database's documents covers 31 of 173 de-centric genetic regulatory targets. The 142 residue de-centric genetic regulatory targets haven't been discovered probably due to the limited experimental techniques. The number of regulators of 31 de-centric genetic regulatory targets is small and not above 5. The ratio of the target's number with equal or below 2 regulators to the total target's number is 0.84 (Figure 6). Hence, SLDR is able to detect the de-centric genetic regulatory targets confirmed by the Yeastract Database.

### 3. Construction of de-centric target-regulator network

**Figure 7:**
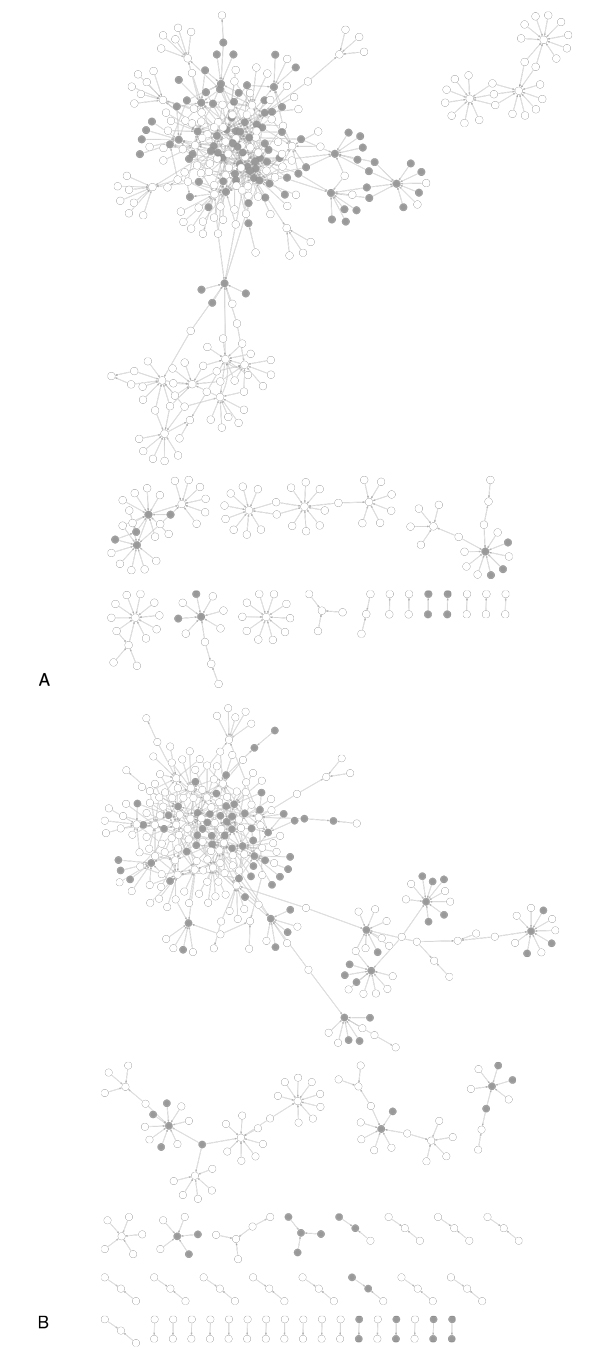
**The de-centric targets and the discovered de-centric regulatory genes in a network shown with only genetic activation and inhibition regulatory relationships**. All the genes from identified genetic regulations (shown as nodes) are colored dark gray, while the new genes of genetic regulation (also shown as nodes) are transparent. The direction of each arrow points from genetic regulator to target.

We constructed the network of the 610 activation pairs and 494 inhibition pairs (Figure 7). 112 targets were found to be new candidates with its genetic regulator. The hub nodes with high-connectivity linked to the majority of nodes to form a main structure of network. The peripheral nodes with low-connectivity formed relativity small sub-networks or even one-to-one model. The ratio of the size of the largest sub-network that exists in this network to the size of this network, we defined as index of aggregation, reflect hub-nodes weight. The index of aggregation in the activation genetic regulatory relationship is 64%. The index of aggregation in the inhibition genetic regulatory relationship is 62%.

### 4. Significance evaluation of de-centric genetic regulatory relationship identified

**Figure 8:**
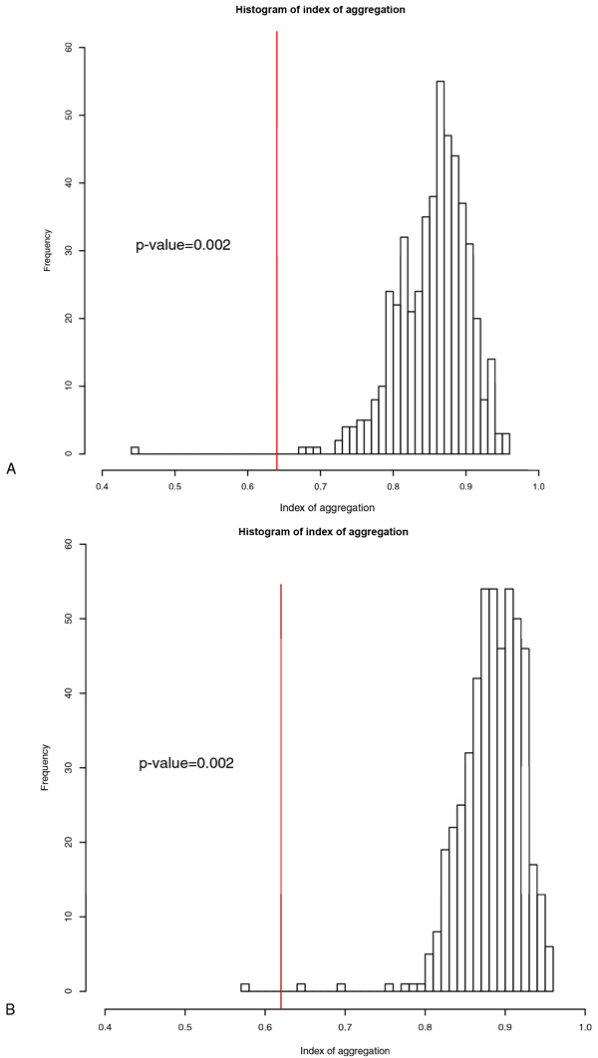
**A histogram showing index of aggregation (IoA) for the decentric genetic regulatory network identified using SLDR**. A) A histogram of the IoA distribution randomly sampled to determine the significance of activation relationship network (size = 83). The red line indicates the IoA = 0.64 for activation type of genetic regulatory network. B) A histogram of the IoA distribution randomly sampled to determine the significance of activation relationship network (size = 93). The red line indicates the IoA = 0.62 for inhibition type of genetic regulatory network.

The empirical distribution of the index of aggregation was obtained after 500 random re-samplings (Figure 8). Only 1 run out of 500 resulted in an index of aggregation value greater than 99.8% in both de-centric genetic activation regulatory network and de-centric genetic inhibition regulatory network. Therefore, the p-value of the index of aggregation is 0.002. It is not surprising to observe such a significant result since the results are selected in a way that the theoretical genetic regulator is equal or below 2. Hence, the aggregation test confirmed result of clustering of gene expression profiles and transcription factor binding site pattern matching is significant in discovering de-centric genetic regulatory relationship.

### 5. Robustness of the genetic regulatory network

After we introduced the noise by randomly changing 5%, 10%, 15%, 20%, 30%, 50%, 70% of total samples, the p-value of network aggregation increase significantly (Table 5). The aggregation p-value below 0.05 is high significant with bold type. The aggregation p-value below 0.1 and above 0.05 is significant with bold type. The aggregation p-value above 0.1 is weak significant or no significant.

**Table 5:** Effect of introducing noise in the results on the network index of aggregation (showing p-value changes)

Noise		0	5	10	15	20	30	50	70
positive	regulator	59	36	20	22	9	7	6	3
	
	p-value	0.002**	0.042**	0.216	0.61	0.294	0.14	0.242	0.292
	
	Variance	0.0025	0.0166	0.0313	0.0174	0.0216	0.0201	0.0188	0.019913

negtive	regulator	48	36	19	17	9	4	4	4
	
	p-value	0.002**	0.014**	0.078*	0.124	0.088*	0.496	0.504	0.404
	
	Variance	0.0016	0.0091	0.0254	0.0196	0.0277	0.0188	0.0145	0.014751

The result shows that the network has resistance of 5% noise. However, along the noise increase, a significant weakening appears on detecting the de-centric regulators and finding potential targets using SLDR. In the randomly generated networks by small number of targets, the network aggregation test tends to be bias reflected on the increased variance.

### 6. Decisive cluster of samples

**Figure 9:**
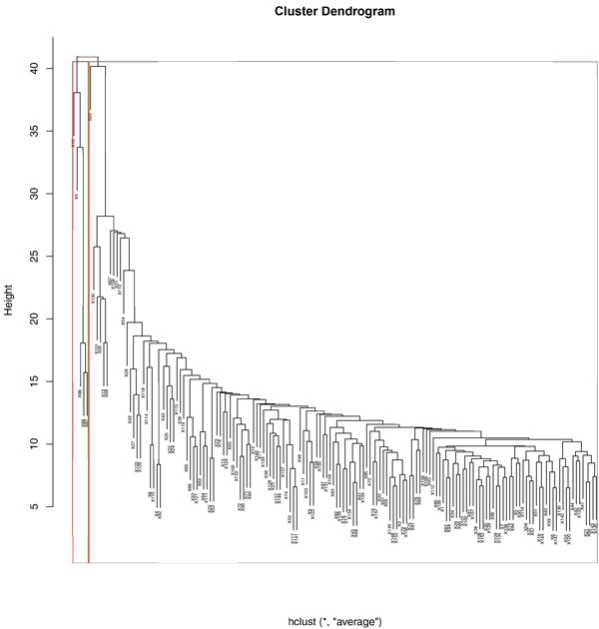
**Hierarchical clustering of all the samples using UPGMA**. The red and green outlined rectangles present two clusters separated by the minimal threshold in the hierarchical tree. The red rectangle includes 5 samples of *ark1*-del+*prk1*-del, *cdk8*-del, *ptc1*-del+*pph3*-del, *ptc1*-del+*ptc2*-del and *ptc1*-del strains. The green rectangle includes residue samples ("All-5 samples").

Applying UPGMA clustering, the samples were divided into two groups using a minimal threshold in the hierarchical tree. We chose the majority of samples which has similarity conditions clustered in green rectangle (Figure 9) and generated the de-centric genetic regulatory targets and pairs applying SLDR method. After we constructed the network of the 999 activation pairs to targets and 805 inhibition pairs,163 targets were found to be new candidates with its genetic regulator. The index of aggregation in the activation genetic regulatory relationship is 78%. The index of aggregation in the inhibition genetic regulatory relationship is 81%.

According to the Pearson Correlation model of Activation/Inhibition, 132 targets with 8558 activation pairs were selected in initial 744 de-centric regulatory targets generated from SLDR and 135 targets with 4486 inhibition pairs were selected in initial 744 de-centric targets generated from SLDR. After ordering all regulation pairs in the genome for each target by finding max *f*(*t, x*): |R_o_-R_h_| and choosing top 10 high pairs, 545 activation pairs and 482 inhibition pairs were selected. (Index 2) 268 targets candidates were queried in Yeast Fitness Database, 105 targets were identified afterwards. Among them, 70 targets with 244 activation pairs and 35 targets with 110 inhibition pairs were certificated. Comparing to the previous genetic regulatory de-centric pairs generated from un-delete samples, the validated activation pairs increase 129( i.e. 5%) while validated inhibition pairs decrease 13 (i.e. 6%). (Table 6)

**Table 6:** Validated de-centric targets and genetic regulatory relationship

	activation			inhibition		
	**validated**	**total**	**rate**	**validated**	**Total**	**rate**

Pairs	115	610	19%	97	494	20%

pairs(after cluster)	244	999	24%	110	805	14%

Change	(+)129	(+)389	(+)5%	(-)13	(-)311	(-)6%

The empirical distribution of the index of aggregation was obtained after 500 random re-samplings. 93 runs out of 500 resulted in an index of aggregation value greater than 81.4% in de-centric genetic activation regulatory network and de-centric genetic inhibition regulatory network and 58 runs out of 500 resulted in an index of aggregation value greater than 88.4% in de-centric genetic activation regulatory network and de-centric genetic inhibition regulatory network. Therefore, the p-value of the index of aggregation in de-centric genetic activation regulatory network is 0.186 and the p-value of the index of aggregation in de-centric genetic inhibition regulatory network is 0.116.

The result (Table 7) shows that the de-centric regulatory network generated without 5 samples of *ark1*-del+*prk1*-del, *cdk8*-del, *ptc1*-del+*pph3*-del, *ptc1*-del+*ptc2*-del and *ptc1*-del strains would entirely lose ability to detect the de-centric targets. In complete samples SLDR method, the network has 5% resistance of noise. Here we selected 5 samples that is 3% of entire samples deletion. Then the aggregation cannot detect de-centric targets. It reveals that the cluster of 5 samples of *ark1*-del+*prk1*-del, *cdk8*-del, *ptc1*-del+*pph3*-del, *ptc1*-del+*ptc2*-del and *ptc1*-del strains is a decisive cluster.

**Table 7:** A Comparison between all the samples and the All-5 Samples (5 deleted after hierarchical clustering) to show changes of p-values for the network's index of aggregation.

noise		total samples	5 samples deletion
positive	regulator	59	132
	
	p-value	0.002^**^	0.186
	
	variance	0.002532727	0.002405613

negative	regulator	48	135
	
	p-value	0.002^**^	0.116
	
	variance	0.001573338	0.006110383

**Figure 10:**
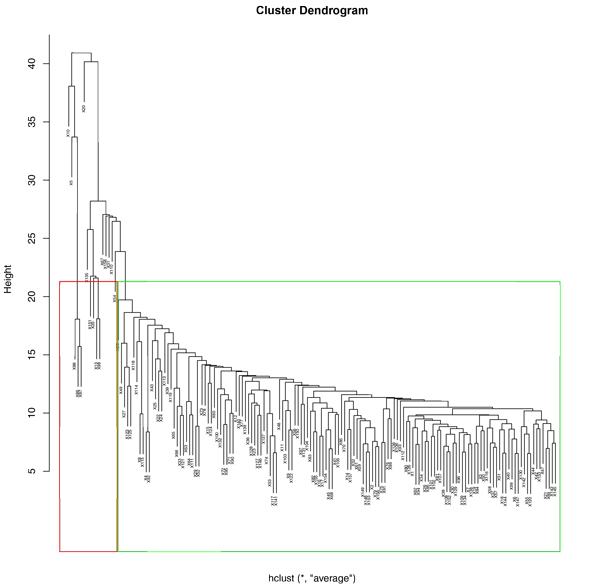
**Hierarchical clustering of ALL samples using UPGMA**. The red and green outlined rectangles present two clusters separated by the small threshold that provide more similarity condition of samples in the hierarchical tree. 14 samples in the red rectangle are not closely related to the cluster in green rectangle.

**Figure 11:**
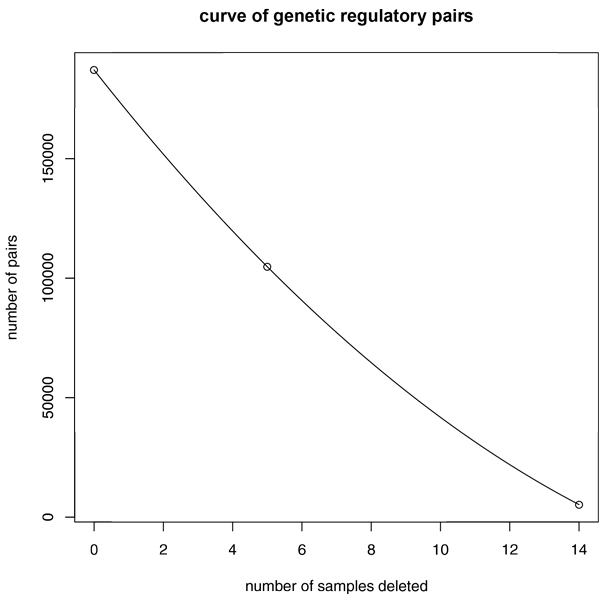
**The inverse relationships between the number of sample used/deleted on the x-axis and the number of significant genetic regulatory relationship pairs identified**. The curve shows that with deletion of important samples for ALL, ALL-5, and ALL-14 data points, significantly less number of genetic relationships maybe found.

In order to see the decisive samples effect on regulatory relationship, we lifted the threshold in the UPGMA hierarchical tree. We chose the more strict similarity of samples which were clustered in green rectangle and deleted the decisive cluster of 16 samples in red rectangle (Figure 10). However, we generated the de-centric genetic regulatory targets and pairs applying SLDR method in the result of only 4 activate genetic regulatory targets with 6 pairs and 3 inhibit genetic regulatory targets with 9 pairs. Hence, the decisive cluster also has effect on finding de-centric pairs, which confirmed by a significant sharp drop in finding the genetic regulatory pairs of regulators to targets by deletion of 16 samples (Figure 11).

### 7. The biology process groups of de-centric genetic regulatory networks

**Figure 12:**
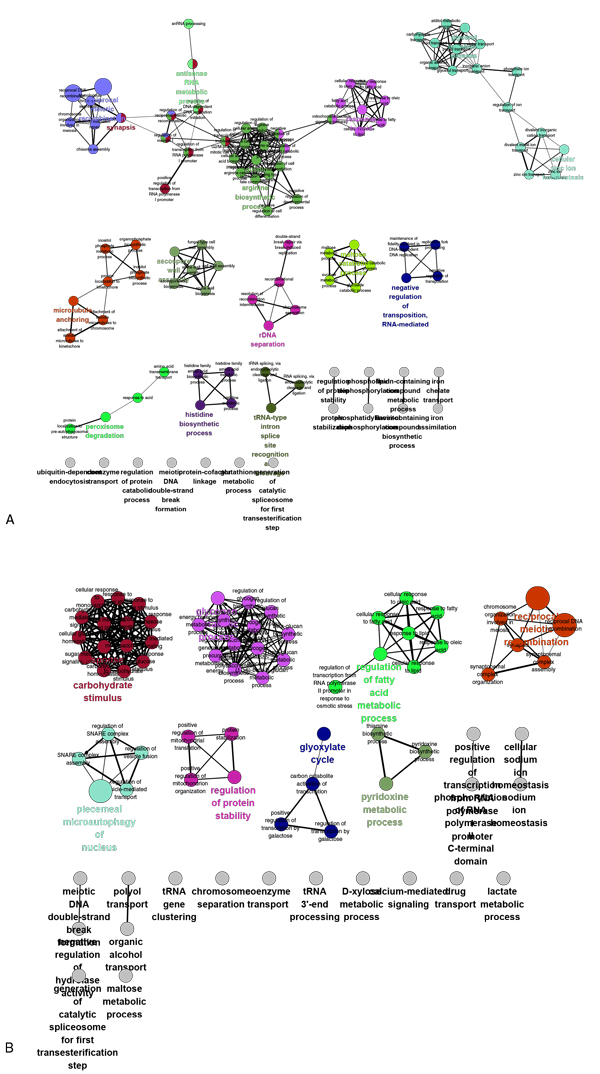
**The biology process groups of de-centric genetic regulatory networks**. A) The biology process groups of genetic activation regulatory networks. B) The biology process groups of genetic activation regulatory networks. The colorful nodes stand for biology processes. The biology processes grouped together with same color by measuring the similarity using Kappa scores. The groups consist of grey nodes were not found a certain and consensus function.

The biology process of de-centric genetic regulatory networks were explored and grouped by ClueGo (Figure 12). In the de-centric activation genetic regulatory network, 14 functional groups were found (Table 8A). 4 significant function with above 10 gene numbers in the groups: synapsis, reciprocal meiotic recombination, glycerol metabolic process, and regulation of arginine biosynthetic process. In the de-centric inhibition regulatory network, 7 functional groups were found (Table 8B). 2 significant function with above 10 gene numbers in the groups: piecemeal microautophagy of nucleus, and reciprocal meiotic recombination. These 5 (3 in activation, 1 in inhibition, 1 in common) significant function indicate that the de-centric genetic regulatory networks were functional and involved in basic and fundamental biology process in cells, especially like reciprocal meiotic recombination, which found in both de-centric activation and inhibition networks.

**Table 8:** The biology process group lists of de-centric genetic regulatory networks.

A.		
**Function**	**Groups**	**Gene numbers**

ascospore wall assembly	Group0	6
peroxisome degradation	Group1	8
**synapsis**	**Group2**	**11**
tRNA-type intron splice site recognition and cleavage	Group3	2
**reciprocal meiotic recombination**	**Group4**	**20**
histidine biosynthetic process	Group5	2
maltose catabolic process	Group6	3
**glycerol metabolic process**	**Group7**	**15**
**regulation of arginine biosynthetic process**	**Group8**	**16**
antisense RNA metabolic process	Group9	6
cellular zinc ion homeostasis	Group10	9
negative regulation of transposition, RNA-mediated	Group11	3
rDNA separation	Group12	8
microtubule anchoring	Group13	5
response to lipid	Group14	7

B.		

**Function**	**Groups**	**Gene numbers**

pyridoxine metabolic process	Group0	3
regulation of fatty acid metabolic process	Group1	4
**piecemeal microautophagy of nucleus**	**Group2**	**10**
glyoxylate cycle	Group3	5
regulation of protein stability	Group4	3
**breciprocal meiotic recombination**	**Group5**	**12**
glycogen metabolic process	Group6	8
response to carbohydrate stimulus	Group7	2

### 8. The sub cellular location of de-centric regulators and targets in cell

**Figure 13:**
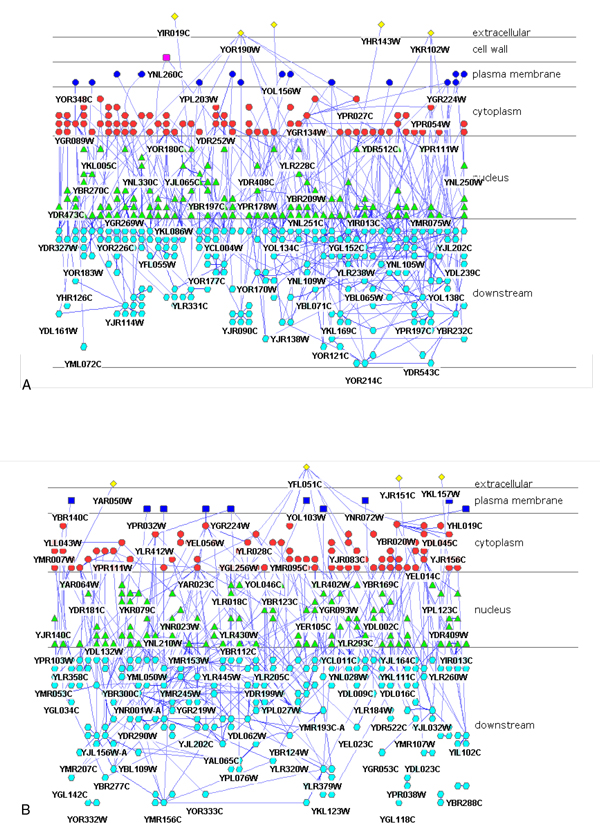
**The sub cellular location of de-centric regulators and targets**. A) The sub cellular location of genetic activation regulatory networks. B) The sub cellular location of genetic inhibition regulatory networks. The regulators and targets were assigned to different layer with distinguishing shapes and colors in cell from extracellular layer to downstream layer. Since the overlap of the name, we only showed some of the gene's name and provide the overview of the location.

The sub cellular location of de-centric regulators and targets showed a complex regulatory networks through the cells (Figure 13). In both de-centric activation regulators and targets networks, and de-centric activation regulators and targets networks, the regulatory pathways signals were cascade in cell - extracellular proteins and membrane receptors at the top, adapter proteins in the cytoplasm, and nuclear proteins and pathway-regulated genes at the bottom.

## Discussion

In this study, we discovered 112 significant new genetic regulatory targets with functional genomics data set derived from 158 viable protein kinase/phosphatase deletion experiments. For each new target, top 10 high-ranking suspected genetic regulatory pair candidates are also given. This genetic regulatory relationship of regulators to targets can include much more than the direct regulation and control, because genetic regulators could sit in the far upstream of its targets.

We developed SLDR to identify candidate genetic regulators particularly of none-hub gene targets. Unlike a whole-transcriptome profiling based correlation techniques, SLDR can efficiently detect signal levels as "clustered conditions", each of which correspond to a "step-level" of target gene perturbed/regulated by a set of combinatorial states of regulator genes. Because the correlation is performed at the "step levels", SLDR can therefore screen, identify, and rank potential regulation relationship of genetic regulators to targets. The prediction pairs can be validated reasonably well by data from the Yeast Fitness Database, although the overlap is not high due to incomplete coverage of the database. Note that our method of finding genetic regulatory relationship of regulators to targets relies on Pearson Correlation at the moment. Future extension of this work could explore additional correlation techniques such as using Goodman and Kruskal's gamma correlation coefficient or Spearman's rank correlation coefficient to compare their impact on prediction performance.

In summary, our study demonstrated a new direction to identify genetic regulatory relationships. If applied broadly, the technique could yield many new data worth biological investigations. Such relationships, although indirect, may help construct biological signaling network to overcome coverage issues facing network biology models today. Future directions to make SLDR an easy-to-use software package and develop databases to capture the results are under development. By applying SLDR to human gene expression perturbation data, we believe our framework may also be extended to provide complementary insights on human complex biological systems and disease network biology.

## Competing interests

Jake Y. Chen is the founder and chairman of Medeolinx, LLC of Indianapolis, IN. All authors declare no competing interests in connection with this work.

## Supplementary Material

Additional file 1 Click here for file

Additional file 2 Click here for file
